# Exercise as a diagnostic and therapeutic tool for preventing cardiovascular morbidity in breast cancer patients– the BReast cancer EXercise InTervention (BREXIT) trial protocol

**DOI:** 10.1186/s12885-020-07123-6

**Published:** 2020-07-14

**Authors:** Stephen J. Foulkes, Erin J. Howden, Yoland Antill, Sherene Loi, Agus Salim, Mark J. Haykowsky, Robin M. Daly, Steve F. Fraser, Andre La Gerche

**Affiliations:** 1 Sports Cardiology Lab, Clinical Research Domain, Baker Heart and Diabetes Institute, 75 Commercial Rd, Melbourne, VIC 3004 Australia; 2 Institute of Physical Activity and Nutrition, School of Exercise and Nutrition Sciences, Deakin University, Geelong, VIC Australia; 3 Melbourne Cancer Care, Cabrini Health, Brighton, VIC Australia; 4 Central Clinical School, Faculty of Medicine, Nursing and Health Sciences, Monash University, Melbourne, VIC Australia; 5 Translational Breast Cancer Genomics Laboratory, Peter MacCallum Cancer Centre, Melbourne, VIC Australia; 6 Department of Population Health, Baker Heart and Diabetes Institute, Melbourne, VIC Australia; 7 Melbourne School of Populatoin and Global Health; School of Mathematics and Statistics, The University of Melbourne, Melbourne, VIC Australia; 8 Faculty of Nursing, University of Alberta, Edmonton, AB Canada; 9 National Centre for Sports Cardiology, St Vincent’s Hospital Melbourne, Melbourne, VIC Australia

**Keywords:** Cardiotoxicity, Exercise training, Anthracycline, Cardiac reserve

## Abstract

**Background:**

Anthracycline chemotherapy (AC) is an efficacious (neo) adjuvant treatment for early-stage breast cancer (BCa), but is associated with an increased risk of cardiac dysfunction and functional disability. Observations suggest that regular exercise may be a useful therapy for the prevention of cardiovascular morbidity but it is yet to be interrogated in a large randomised trial.

The primary aims of this study are to: *1)* determine if 12-months of ET commenced at the onset of AC can reduce the proportion of BCa patients with functional disability (peak VO_2_, < 18 ml/kg/min), and *2)* compare current standard-of-care for detecting cardiac dysfunction (resting left-ventricular ejection fraction assessed from 3-dimensional echocardiography) to measures of cardiac reserve (peak exercise cardiac output assessed from exercise cardiac magnetic resonance imaging) for predicting the development of functional disability 12-months following AC. Secondary aims are to assess the effects of ET on VO2peak, left ventricular morphology, vascular stiffness, cardiac biomarkers, body composition, bone mineral density, muscle strength, physical function, habitual physical activity, cognitive function, and multidimensional quality of life.

**Methods:**

One hundred women with early-stage BCa (40–75 years) scheduled for AC will be randomized to 12-months of structured exercise training (*n* = 50) or a usual care control group (*n* = 50). Participants will be assessed at baseline, 4-weeks following completion of AC (4-months) and at 12-months for all measures.

**Discussion:**

Women diagnosed with early-stage BCa have increased cardiac mortality. More sensitive strategies for diagnosing and preventing AC-induced cardiovascular impairment are critical for reducing cardiovascular morbidity and improving long-term health outcomes in BCa survivors.

**Trial registration:**

Australia & New Zealand Clinical Trials Registry (ANZCTR), ID: 12617001408370. Registered on 5th of October 2017.

## Background

Breast cancer (BCa) is the most commonly diagnosed cancer among women, with over 1.6 million women diagnosed globally each year [1]. Advances in detection and treatment have improved cancer-specific survival such that the 5-year survival rate is now approaching 90% [2, 3]. An unexpected consequence of this success is that early stage BCa survivors are as likely to die of cardiovascular (CV) causes as they are from BCa [4–6]. This may be due to a combination of common cardiac risk factors combined with toxicity from cancer therapies, particularly anthracycline chemotherapy (AC) [7, 8]. Whilst AC is one of the mainstays of neoadjuvant and adjuvant therapy for triple-negative and locally-advanced BCa [9], it induces dose-dependent CV injury causing reductions in functional capacity (measured objectively as a peak oxygen uptake, peak VO_2_, < 18 ml/kg/min) that is associated with an increased risk of heart failure (HF) [10]. AC-mediated cardiac dysfunction shows limited reversibility with pharmacological treatment, particularly if detected late [11]. Furthermore, those who go on to develop symptomatic HF experience poor mortality outcomes [12]. As such there is an emphasis on detecting cardiac dysfunction at the earliest possible stage.

Findings from a meta-analysis indicated that time since treatment is an important risk factor for cardiotoxicity [13]. Indeed, the discrepancy between the rates of cardiac dysfunction detected soon after treatment and long-term heart failure incidence [10] highlights that an absence of measurable cardiac dysfunction soon after treatment does not adequately predict the risk of subsequent toxicity. This also emphasises the need for improved early detection strategies [14, 15]. Currently, the cornerstone for detecting AC-induced cardiac dysfunction is measuring changes in resting left-ventricular ejection fraction (LVEF) [14–17]. Whilst LVEF has been in use for decades, its ability to predict subsequent cardiotoxicity is limited by poor reproducibility [18, 19], load and heart rate dependence, and the current LVEF-based classification for cardiotoxicity (typically a > 10% drop from baseline to a value < 50–53%) shows weak associations with heart failure outcomes [20, 21]. Furthermore, half of HF patients have preserved LVEF (> 50%), highlighting that LVEF is insensitive to clinically significant cardiac dysfunction [22]. Consequently, there is growing interest in alternative measures for early detection of cardiac dysfunction following AC [15, 16].

The assessment of an individual’s VO_2_peak has been recently endorsed by the American Heart Association as an important primary endpoint for individuals with- or at risk of HF [23] as it can capture the degree of impairment along the oxygen cascade [24], whilst providing meaningful information on functional capacity [24, 25], and HF incidence [26, 27], and prognosis [28, 29]. The functional impact of cardiotoxic BCa treatments may be quantified using cardiopulmonary exercise testing as a VO_2_peak below 18.0 mL/kg/min, which is indicative of ‘functional disability’ given its approximation to the level of fitness required to perform simple activities of daily living [25]. This threshold is associated with a 7–9 fold increase in the risk of heart failure [26, 30], and a two-fold increased risk of all-cause mortality in metastatic BCa survivors [31]. Importantly, as many as 29–50% of BCa survivors fall below this threshold despite having a normal resting LVEF [31, 32], highlighting the need for better diagnostic approaches. Some of the key limitations of resting LVEF for predicting functional disability and HF risk could be overcome through the assessment of cardiac reserve, defined as the increase in cardiac function from rest to peak exercise. This is based on the premise that symptoms of HF typically present with minimal levels of exertion, when the heart has insufficient reserve to adequately respond to the demands of exercise. The use of cardiac imaging is advantageous as it provides a specific assessment of cardiac reserve. Whilst posing several technical challenges, the development of novel imaging techniques such as exercise cardiac magnetic resonance imaging (ExCMR) allows for the assessment of biventricular function with a high degree of accuracy [33], and may provide a more meaningful understanding of heart failure risk and functional capacity in BCa survivors than resting LVEF [32].

Current approaches for preventing cardiovascular morbidity in patients receiving anthracyclines include treatment withdrawal and/or modification, and pharmacological strategies. Treatment withdrawal prevents further cardiac injury, however is problematic due to the potential negative effects on cancer-related outcomes [34]. The use of pharmacotherapies such as Dexrazoxane [35, 36], angiotensin converting enzyme inhibitors [35], and beta-blockers [35] can reduce the risk of subsequent cardiac dysfunction. However, this appears at odds with the current trend towards personalized therapy, given that this would result in the majority of patients being treated unnecessarily. Additionally, given cardiac function is unlikely to be the sole driver behind AC-induced impairments in exercise capacity and functional disability [37], the ability of cardiac-focused pharmacotherapy to completely reverse a patient’s exercise intolerance may be limited. Exercise training (ET) has emerged as an important therapeutic tool for addressing a number of adverse effects associated with cancer treatment [38], and there is growing interest in its use for preventing cardiotoxicity and functional disability [39]. However, whilst exercise can prevent or attenuate declines in VO_2_peak during BCa chemotherapy (predominantly anthracycline-based) [38, 40–42], no randomised trials have investigated whether it can reduce the incidence of important clinical endpoints such as functional disability. Furthermore, the degree to which the beneficial effects on VO_2_peak reflects cardiac versus peripheral ‘protection’ is still unknown and will have important implications for the cardioprotective role of exercise. The primary trials investigating the effect of exercise training on cardiac function during AC have shown neither a beneficial, nor detrimental effect on cardiac function [32, 40, 43]. These studies have been small, short-term and the majority have relied on resting measurements of cardiac function to identify cardiac dysfunction. Thus, there is a need for larger, longer RCTs that are based on outcomes that are more sensitive to cardiac dysfunction and prognosis.

Therefore, in women with BCa undergoing anthracycline-based chemotherapy, this 12-month RCT has two primary aims:

1. To compare the current standard-of-care (resting LVEF) to measures of cardiac reserve (peak exercise cardiac output; Qc) as predictors of functional disability
2. To determine whether a 12-month structured exercise training (ET) program reduces the proportion of BCa patients who are functionally disabled 12-months after the initiation of AC.

We hypothesize that:

1. Cardiac reserve will be superior to resting LVEF at predicting the development of functional disability 12-months following AC
2. Participating in a 12-month structured ET will reduce the proportion of patients who are functionally disabled 12-months following AC.

Secondary aims include assessing the effect of ET on changes in cardiopulmonary fitness and cardiac reserve, along with indices of resting cardiac structure and function, vascular stiffness, biochemical and blood-based markers of cardiovascular function, total- and regional body composition, bone mineral density of the lumbar spine and femoral neck, muscle strength, physical function, habitual physical activity, cognitive function, and multidimensional quality of life.

## Methods

### Study design

**Fig. 1:**
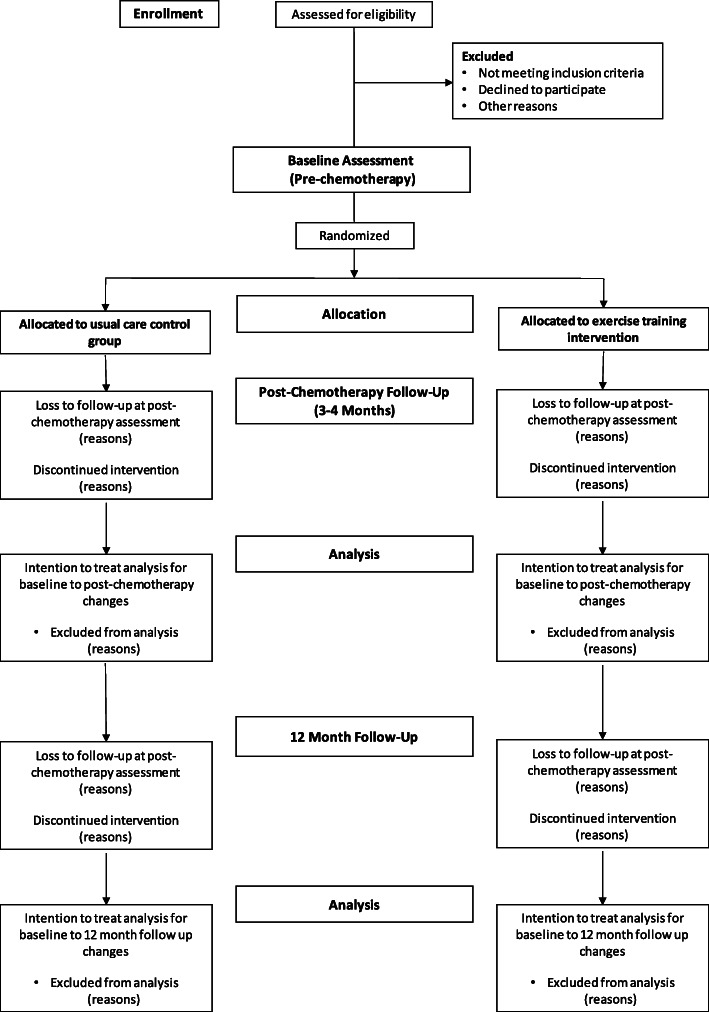
Study CONSORT flow diagram

This study will be a 12-month, community-based, two-arm randomised controlled trial in women with BCa undergoing AC comparing (i) the ability of ExCMR versus resting echocardiography to predict patients who will become functionally disabled following AC; and (ii) the relative effectiveness of a 12-month supervised and structured multi-component exercise program to usual care for preventing functional disability following AC. A total of 100 women with BCa aged 40–75 years who are scheduled to undergo AC will be recruited and randomly allocated to either a 12-month multi-component exercise program (ET, *n* = 50) or a usual care control group (UC, n = 50). All assessments will be performed at the Baker Heart and Diabetes Institute (Melbourne, Victoria, Australia) at baseline (no more than 2-weeks following the commencement of AC), 4-months (~ 3 weeks following the completion of AC) and 12-months from the commencement of AC. A flow diagram of the study protocol is shown in Fig. 1. Where possible, all baseline assessments will be conducted prior to the commencement of AC, however this may not always be possible due to the short time frame between patients being informed of the decision to undergo AC and its commencement. This trial has been approved by the Alfred Hospital Human Research Ethics Committee (Project No: 305/17), is registered with the Australian and New Zealand Clinical Trials Registry (ACTRN12617001408370) and is funded by the World Cancer Research Fund International (Grant IIG_2019_1948).

### Participants

Women deemed eligible to participate in the trial include those aged 40–75 years who have a histologically confirmed diagnosis of breast cancer and are scheduled for anthracycline-based chemotherapy. Participants will be excluded if they have: (1) known structural heart disease including symptomatic ischemic heart disease, significant valvular disease or inherited cardiomyopathies (which would contraindicate AC), (2) a contraindication to CMR such as a pacemaker or implanted metallic foreign body or device, (3) the presence of any serious contraindication or uncontrolled medical condition that would limit participation in the exercise program as outlined in guidelines from the American College of Sports Medicine [44], (4) an inability to complete questionnaires in English language, or (5) significant cognitive impairment (determined by the short portable mental status questionnaire) [45].

### Recruitment and screening

Participants will be recruited via direct referral from surgeons and oncologists from a variety of private and public oncology services around metropolitan Melbourne, Victoria, Australia. Oncology services will be contacted via email with information regarding the study. Group presentations outlining the study rationale, study procedures and eligibility criteria will be organised for oncology services interested in referring potential candidates. Participants identified as potentially eligible by their clinicians will be provided with written material outlining the purpose of the study and requirements of participation prior to being screened over the phone by a member of the research team. Individuals interested in participating will then provide written informed consent after further verbal discussion with a senior investigator.

### Randomisation and blinding

Following baseline testing, each participant will be randomly allocated (1:1 ratio) to the intervention or control group by an independent researcher using a computer-generated, random number sequence with the outcome communicated via telephone. Stratified block randomisation will be used, with participants stratified by age (< 60 or ≥ 60 years) and human epidermal growth factor receptor 2 (HER2) status (positive or negative), with block sizes alternating between two and four participants. Participants, care providers and outcome assessors will not be blinded to group allocation. However, the quantification of all cardiac imaging (echocardiography and cardiac magnetic resonance imaging, CMR) will be performed by researchers blinded to subject identity. Furthermore, outcome assessors will be blinded to pre-chemotherapy values for all assessments.

### Intervention group

This is a multi-component periodised ET intervention designed to address the negative consequences of AC on cardiac, vascular, and skeletal muscle function. There will be three major phases to the program: Phase 1 - A 12-week structured, supervised exercise program conducted during AC; Phase 2 - A 14-week structured semi-supervised exercise program following AC; and Phase 3 - A 26-week step-down maintenance exercise program.

#### Phase 1 – structured exercise during AC (week 1–12)

The exercise training program conducted during AC will consist of 30–60 min of supervised, multi-modal exercise training performed three times per week. Sessions will use a combination of aerobic and progressive resistance training (PRT) and will be conducted at the Baker Heart and Diabetes Institute, the Deakin University Clinical Exercise Learning Centre, and participating health and fitness centres throughout metropolitan Melbourne. Sessions will be prescribed and overseen by an Accredited Exercise Physiologist (AEP), with all training supervised by appropriately trained AEPs and/or Exercise Scientists. A novel, non-linear step periodization model will be used due to its ability to adjust for fluctuations in each participant’s symptoms throughout their chemotherapy cycles whilst still allowing for adequate progression of training volume [46]. The model used in this study will involve a progressive increase in exercise volume of ~ 5–10% each week until the week immediately following each participant’s chemotherapy cycle. This week will be considered a ‘de-loading’ week where training intensity will be reduced by ~ 5%.

##### Aerobic ET

The aerobic component of the program will consist of both continuous steady state and interval-based training to provide varied forms physiological perturbation to the different components of the oxygen cascade that could be affected by chemotherapy [46]. Interval sessions will be performed on a cycle ergometer, whilst the continuous training will be performed on an upright cycle, treadmill and/or elliptical trainer based on participant preference. Exercise intensity will be individualised from each participant’s percentage of heart rate reserve (%HRR) at their ventilatory threshold (VT) measured during the baseline cardiopulmonary exercise test (CPET). Aerobic exercise intensity will be monitored by the 1–10 rating of perceived exertion (RPE) scale and wrist-worn heart rate (HR) monitors (Polar M200, Polar, Kempele, Finland), and these will be used to adjust the exercise workloads to account for day-to-day variation in participant health status throughout each chemotherapy cycle. The program will be broken into four training blocks based on participant’s scheduled chemotherapy in weeks 0, 3, 6 and 9 with progression of training volume outlined in Table 1. All sessions will include a 5-min aerobic warm up and cool-down. Following a one-week lead in period consisting of 3 sessions of 30-min at an intensity 10–15 beats/min below the VT, participants will complete two steady state aerobic sessions and one vigorous to high intensity interval session per week for the remaining 11 weeks, with progressive increases in exercise duration and/or intensity as outlined in Table 1. Interval sessions will begin in week 2, and consist of four work intervals of 2–4 min progressing from the %HRR corresponding to VT and progressing to 85–95% HR_peak_, interspersed with 3-min of cycling at a light intensity. The target intensity of the continuous and interval training will be reduced by ~ 5% in week 3, 6 and 9 to account for the increased symptom burden of each chemotherapy cycle.

**Table 1:** Progression of the 12-month multi-modal exercise training program

Phase	Cycle	Weeks	Session Type	Frequency (per week)	Duration/Dose	Intensity^**a**^
Phase 1 Supervised Exercise During AC	1	1–3	Steady State & Resistance Training	2	SS: 30 mins RT: 1–2 sets × 12–15 reps	SS: 10–20 b/min below %HRR at VT RT: 60–70% 1RM
			Interval Training	1	4 × 2 mins^b^	%HRR at VT ± 5 b/min
	2	4–6	Steady State & Resistance Training	2	SS: 30 mins RT: 2 sets × 12–15 reps	SS: 10–15 b/min below %HRR at VT RT: 60–70% 1RM
			Interval Training	1	4 × 3 mins^b^	%HRR at VT ± 5 b/min
	3	7–9	Steady State & Resistance Training	2	SS: 30–35 min RT: 2 sets × 18–12 reps	SS: 5–10 b/min below %HRR at VT RT: 70–85% 1RM
			Interval Training	1	4 × 3 mins^b^	85–95% HR_peak_
	4	10–12	Steady State & Resistance Training	2	SS: 35–40 min RT: 2 sets × 8–12 reps	SS: 5–10 b/min below %HRR at VT RT: 70–85% 1RM
			Interval Training	1	4 × 4 mins^b^	85–95% HR_peak_
Phase 2 Semi-supervised Exercise Following AC	1	13,15,17	Endurance Training	1	40–50 min	15–20 b/min below %HRR at VT
			Tempo Training & Resistance Training	2	TT: 35 mins RT: 2 sets × 8–12 reps	TT: 5–10 b/min below %HRR at VT RT: 70–85% 1RM
			Interval Training	1	4 × 4 mins^b^	85–95% HR_peak_
		14,16	Tempo Training	1	35 mins	5–10 b/min below %HRR at VT
			Interval Training & Resistance Training	2	IT: 4 × 4 mins^b^ RT: 2 sets × 8–12 reps	IT: 85–95% HR_peak_ RT: 70–85% 1RM
			Recovery Session	1	30 mins	25–30 b/min below %HRR at VT
	2	18,20,22	Tempo Training	1	35 mins	0–5 b/min below %HRR at VT
			Interval Training & Resistance Training	2	IT: 4 × 4 mins^b^ RT: 2 sets × 8–12 reps	IT: 85–95% HR_peak_ RT: 70–85% 1RM
			Recovery Session	1	30 mins	20–25 b/min below %HRR at VT
		19,21	Endurance Training	1	50–60 min	15–20 b/min below %HRR at VT
			Tempo Training & Resistance Training	2	TT: 35 mins RT: 2 sets × 8–12 reps	TT: %HRR at VT ± 5 b/min RT: 2 sets × 8–12 reps
			Interval Training	1	4 × 4 mins^b^	85–95% HR_peak_
	3	23,25	Endurance Training	1	60 mins	10–20 b/min below %HRR at VT
			Tempo Training & Resistance Training	2	TT: 35 mins RT: 2 sets × 8–12 reps	TT: %HRR at VT ± 10 b/min RT: 70–85% 1RM
			Interval Training	1	4 × 4 mins^b^	85–95% HR_peak_
		24,26	Tempo Training	1	35 mins	%HRR at VT ± 10 b/min
			Interval Training & Resistance Training	2	IT: 4 × 4 mins^b^ RT: 2 sets × 8–12 reps	IT: 85–95% HR_peak_ RT: 70–85% 1RM
			Recovery Session	1	30 mins	20–25 b/min below %HRR at VT
Phase 3 Maintenance	n/a	27–52	Endurance Training	1	60 mins	10–20 b/min below %HRR at VT
			Tempo Training & Resistance Training	2	TT: 35 mins RT: 2 sets × 8–12 reps	TT: %HRR at VT ± 10 b/min RT: 2 sets × 8–12 reps
			Interval Training	1	4 × 4 mins^b^	85–95% HR_peak_

*Abbreviations*: *%HRR* Percentage of heart rate reserve, *1RM* One repetition max, *HR*_*peak*_ Heart rate peak, *IT* Interval training, *RT* Resistance training, *TT* Tempo training, *VT* Ventilatory threshold

^a^Intensity reduced by 5% from values reported in table during the week following chemotherapy administration

^b^Only duration for work phase of intervals is reported – duration for recovery phase was 3 min of light-intensity cycling

##### Progressive resistance training

For two of the three weekly sessions, participants will also complete six compound PRT exercises (three upper body, three lower body) with a primary focus on improving muscle strength and muscle mass. The PRT exercises will be performed for 1–2 sets of 8–15 repetitions depending on the training cycle (outlined in Table 1). Examples of the exercises to be incorporated in the program include leg press, squats, lunges, step-ups, chest press, overhead press, seated row, and latissimus dorsi pulldown. During the first 6-weeks of the program, participants will perform 1–2 sets of 12–15 repetitions at 60–70% of their one repetition maximum (1RM) strength with 1 min of rest in between each set. During the weeks 7–12 of the program, participants will perform 2 sets of 8–12 repetitions at 70–85% of their 1RM with 1–2 min rest in-between each set. All participants will be instructed to lift and lower the weight in a slow- and controlled manner. Resistance exercises performed in weeks 1–6 will be changed or slightly modified during weeks 7–12 to provide training variety, and progression.

#### Phase 2 – structured semi-supervised ET following AC (week 13–26)

During phase 2, the same personalised, structured exercise program will be prescribed but with an increase in total exercise frequency to four sessions per week. To encourage increased independence there will be a reduced frequency of supervision (twice per week), with the remaining two sessions performed by the participants without supervision. Unsupervised sessions will be completed at each participant’s local health and fitness center or as a home-based exercise session depending on participant preference. During the supervised sessions, participants will receive feedback and guidance on structuring and performing their independent exercise sessions in order to increase their exercise self-efficacy. During phase 2, there will be an emphasis from AEPs on motivational interviewing and goal setting to assist participants in incorporating a regular exercise routine into their lifestyle and to assist in the transition to phase 3 of the exercise program. During week 5 and 10 of the phase 2 program, participants will have a de-loading week, which consists of a 10% reduction in aerobic exercise intensity and a reduction to 1 set of each resistance exercise, thereby facilitating an opportunity for recovery and adaptation.

##### Aerobic ET

**Fig. 2:**
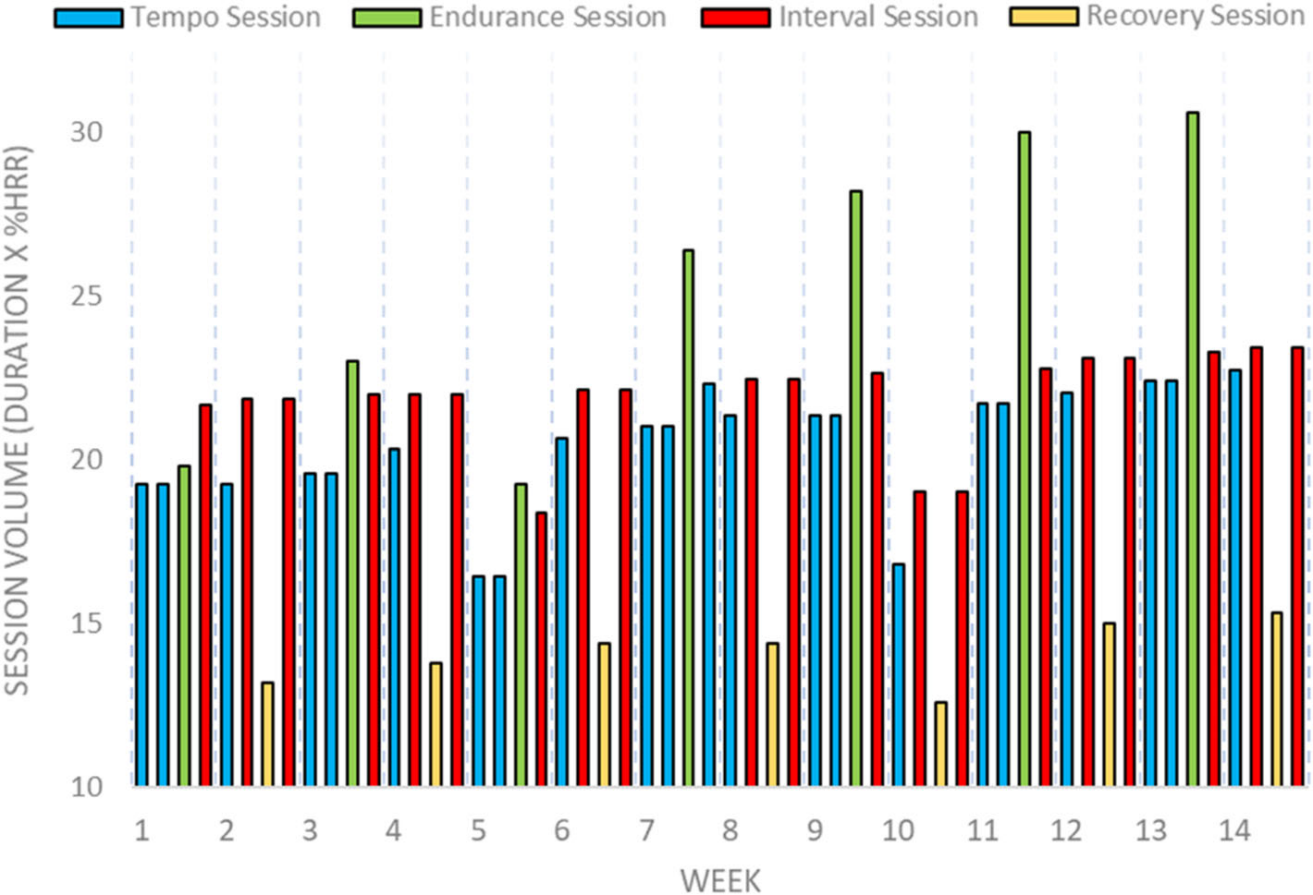
Progression of aerobic exercise training volume during phase 2 of the exercise intervention. Participants complete four sessions per week consisting of a combination of tempo (blue), endurance (green), interval (red) and recovery sessions (yellow) which progress in volume each week over the 16-week training period. A de-load week (10% reduction in exercise intensity) is completed in weeks 5 and 10 to facilitate adaptation and recovery

During phase 2, participants will complete four sessions per week of aerobic training. The aerobic training program completed during phase 2 will consist of four session types: maximal steady state, endurance, interval and recovery sessions (outlined in Table 1 and Fig. 2) that alternate in a bi-weekly cycle similar to previous work in middle-aged adults shown to improve fitness and cardiovascular function [47]. In the first week, participants will complete two tempo sessions, one endurance session, and one interval session. In the alternate week, participants will complete one tempo, and two interval sessions that are interspersed with a recovery session. Tempo sessions will consist of 35 min at the %HRR corresponding to VT ± 10 beats/min as measured from the follow-up CPET at the 4-month testing visit. Endurance sessions will begin with 40-min at the %HHR 10–20 beats/min below VT, and progress by 5-min every fortnight until participants are completing a total duration of 60-min. The interval sessions will be identical to those completed at the end of Phase 1 of the program (4-min intervals at 85–95% HR_peak_). During weeks that incorporate two interval sessions, these sessions will be interspersed with a recovery session consisting of 30-min at an intensity 20–30 beats/min below %HRR at VT.

##### Progressive resistance training

Participants will continue with the same PRT format of 2 sets of 6 exercises at 8–12 RM with 1–2 min rest between sets.

#### Phase 3 – step-down maintenance program (week 27–52)

During phase 3 of the exercise program, participants will continue to follow the same exercise program completed at the end of Phase 2, with adaptations from the study AEP so that they can complete the program independently at home and/or within their community health and fitness centre. Participants will be provided with ongoing support via weekly text reminders from the Physitrack mobile app, and six face-to-face review appointments with the study AEP. Review appointments will be used for goal setting, behavioural counselling and to progress the exercise program. The timeframe of the review sessions will be based on each participant’s preferences and the schedule of their other cancer treatments.

### Usual care group

Participants allocated to usual care will receive ongoing care from their oncology team but will not receive additional access to supervised exercise training from the research team. Control group participants will receive usual lifestyle advice as part of their routine clinical care in which patients will be provided a copy of the Cancer Council Australia booklet entitled “Exercise for People Living with Cancer.” Exercise will then be left to the patient’s volition, including any decision to enrol in a structured exercise program. A sham exercise comparator group will not be used because our primary outcome is an objective, measurable endpoint that is not subjected to patient expectancy or placebo effects.

### Measurements

All measures will be collected at baseline (within 2 weeks of the initiation of AC), following the completion of AC (4-months) and again at 12-months following the initiation of AC. Assessments will be performed at the Baker Heart and Diabetes Institute clinical research facility over two non-consecutive days. Testing session 1 will be conducted prior to chemotherapy, whilst it will be the aim to complete session 2 within the first 2 weeks of starting AC. Session 1 will consist of the resting echocardiography and blood pressure, cognition testing, questionnaires, CPET, blood sample, ExCMR and training in the use of the accelerometer devices for measurement of habitual physical activity. Tests completed during session 2 will include strength and physical function testing and dual-energy x-ray absorptiometry (DXA) scanning.

### Primary and secondary outcome measures

The primary outcome for this study will be the prevalence of functional disability (defined as VO_2_peak ≤ 18.0 mL/kg/min) measured via CPET at 12 months. The predictive ability of standard-of-care versus novel cardiac reserve measures will be addressed by comparing LVEF assessed via 3-dimensional (3D) echocardiography to cardiac reserve assessed via exCMR. For the purposes of this study, impaired cardiac reserve will be defined as a < two-fold increase in Qc from rest to peak exercise [33]. Impaired LVEF will be defined as a LVEF < 53% which is in line with current cardio-oncology guidelines [14, 15, 17].

Secondary outcomes will include changes in cardiopulmonary fitness and cardiac reserve, along with indices of resting cardiac structure and function, vascular stiffness, biochemical and blood-based markers of cardiovascular function, total- and regional body composition, bone mineral density of the lumbar spine and femoral neck, muscle strength, physical function, habitual physical activity, cognitive function, and multidimensional quality of life.

Additional exploratory outcomes will include the association between changes in cardiopulmonary fitness with indices of cardiac (cardiac reserve) versus non-cardiac factors (central vascular stiffness, haemoglobin concentration, lower body lean body mass, skeletal muscle composition of the thigh). The study will also explore the effect of the intervention on treatment-related variables including the dose of treatment received and response to neoadjuvant therapy.

### Cardiopulmonary fitness and functional disability

Cardiopulmonary exercise testing will be used to assess VO_2_peak and functional disability. VO_2_peak, VT and ventilatory efficiency (Minute ventilation to carbon dioxide production slope [VE/VCO_2_ slope]) will be assessed using a continuous ramp protocol on an electronically braked upright cycle ergometer (Lode Excalibur Sport, Lode BV Medical Technology, Groningen, NL) with breath-by-breath expired gas analysis (Vyntus™ CPX, CareFusion, San Diego, CA) in accordance with published guidelines [48]. A flow meter and gas analyser calibration will be performed prior to each test in accordance with the manufacturer guidelines. Two minutes of resting data will be collected prior to the start of exercise, after which participants will undertake a one-minute warm-up at 10–25 W. The workload then increases at a continuous rate of 5–25 W/min until volitional fatigue or symptom limitation. The protocol will be individualised based on each participant’s self-reported physical activity levels, with the aim of reaching volitional exhaustion by 8–12 min. HR and rhythm will be monitored continuously throughout exercise using a 12-lead ECG (Vyntus™ CPX, CareFusion, San Diego, CA) and blood pressure (BP) will be measured every 2 min using an automated cuff (Tango® M2 ECG-gated Automated Blood Pressure Monitor, SunTech Medical Inc., Morrisville, NC). For the purposes of analysis, the test will be considered a peak effort if two of the following criteria are reached: 1) volitional exhaustion; 2) a respiratory exchange ratio > 1.1, and/or 3) > 85% of age-predicted maximal HR [48]. VO_2_peak is defined as the highest 30-s rolling average calculated from six consecutive 5-s VO_2_ epochs. Functional disability will be defined as a VO_2_peak ≤ 18.0 mL/kg/min ref. VT will be assessed using the V-slope method, and the relative proportion of VO_2_peak at which the VT occurs will be used as a measure of changes in submaximal exercise capacity. VE/VCO_2_ slope will be obtained from linear regression analysis of minute ventilation (VE) and expired carbon dioxide (VCO_2_) from the end of the warm-up to the VT [48]. HR and blood pressure (BP) recovery will also be assessed at 1, 2 and 4 min after the end of the test as markers of autonomic function.

### Cardiac reserve

Cardiac reserve will be quantified using exCMR. The real-time CMR protocol used in this study has been described in detail previously and validated against invasive measures [33]. In brief, imaging will be performed with a Siemens MAGNETOM Prisma 3.0 T CMR with a 5-element phased array coil. Ungated real-time steady state free-precision cine imaging will be performed without cardiac or respiratory gating. Using this technique, our group has demonstrated excellent interobserver (R = 0.98 and R = 0.97 for LV and RV SV, respectively) and interstudy reproducibility (R-0.98 for Qc) [33].

**Fig. 3:**
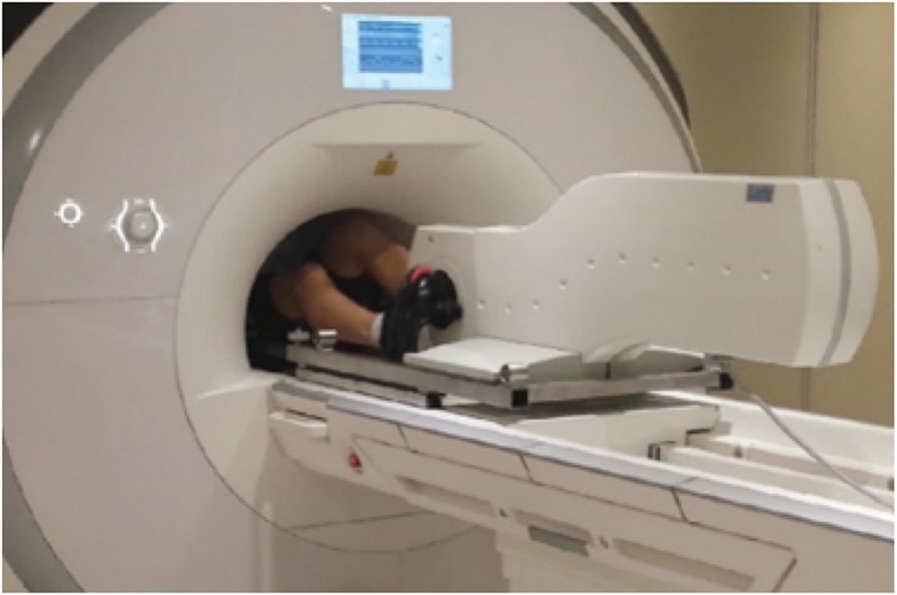
Exercise is performed within the MRI scanner (top image) using the Lode MR Ergometer Pedal with images acquired in real-time during exercise. Exercise is performed at workloads individualised from each participant’s peak workload from their upright cardiopulmonary exercise test

After resting images have been obtained, subjects will cycle on an ergometer compatible for magnetic resonance imaging ([MRI]; MR Ergometer Pedal, Lode, Groningen, Netherlands – Fig. 3) at an intensity equal to 20, 40 and 60% of maximal power output obtained during the upright incremental CPET. These workloads will subsequently be referred to as rest and low, moderate, and high intensity. It has been previously determined that 66% of the maximal power during upright cycling approximates maximal exercise capacity in a supine position for non-athletes [49, 50]. Each stage of exercise is maintained for up to 1.5–3 min; approximately 30 s to achieve a physiological steady-state and 1–2.5 min for image acquisition.

**Fig. 4:**
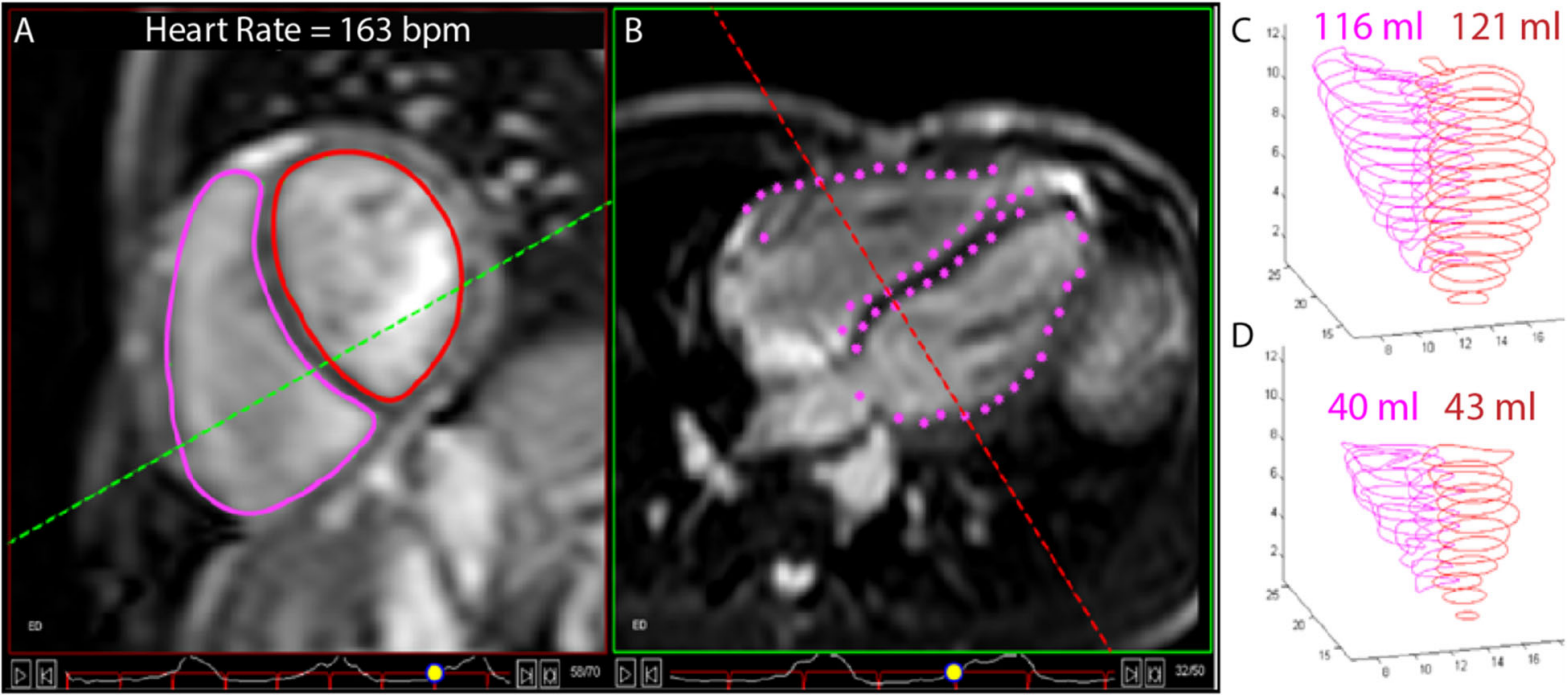
Example of real-time ungated exercise cardiac MRI imaging during high-intensity exercise. **a** Short axis images are used to define the endocardial borders for the calculation of ventricular volumes. The point at which these transect the horizontal long-axis plane (**b**) is shown by the pink dots at the line of the red dotted line. This allows for cross-checking for the accuracy of endocardial contours and for the determination of the atrio-ventricular level on the short axis images. The endocardial ventricular borders for each short-axis slice at (**c**) end-diastole and (**d**) end-systole are summed to determine end-diastolic and end-systolic ventricular volumes respectively. This process is performed for images taken at rest and all intensities of exercise

Images will be analysed on a software program developed in-house (RightVol – Right Volume Leuven, Leuven, Belgium) in which the physiological data (respiratory movement and ECG) are synchronized to the images so that contouring can be performed at the same point in the respiratory cycle thereby greatly minimizing cardiac translation error Fig. 4. Left ventricular (LV) and right ventricular (RV) endocardial contours will then be manually traced on the short axis image, and the points of transection with the horizontal long axis plane are indicated, thus enabling constant referencing of the atrioventricular valve plane. Trabeculations and papillary muscle will be considered part of the ventricular blood pools and volumes will be calculated by a summation of disks (Fig. 3). SV will be calculated from the difference between end-diastolic volume and end-systolic volumes, while Qc will be calculated as (RVSV+LVSV/2) × HR. Peripheral muscle arterio-venous oxygen extraction will be estimated according to the Fick principle [51], using V̇O_2_peak measured by CPET and peak Qc measured by exercise CMR with adjustment for changes in haemoglobin concentration. Cardiac reserve will be defined as the change in Qc from rest to the high intensity workload. This study will also assess changes in HR, SV, LVEF and RVEF at each stage of exercise (rest, low, moderate and high intensity workloads) as additional measures of cardiac reserve.

### Cardiac structure and function

#### Echocardiography

Resting RV and LV function will be assessed by a comprehensive resting echocardiogram (Vivid E95, General Electric Medical Systems, Milwaukee, Wisconsin) with images analysed using offline analysis software (Echopac v13.0.00, GE, Norway). Resting echocardiography represents the current clinical standard of care to which exCMR will be compared [15]. LVEF will be used as the primary ‘standard of care’ measurement, and will be quantified from a full-volume 3D dataset according to standard recommendations. Additional measurements performed will include Doppler, torsion, global longitudinal strain and strain rate measurements.

#### Cardiac magnetic resonance imaging

In addition to resting echocardiography, resting CMR (using the same protocol as described previously) [52] will be used to provide a highly accurate and comprehensive characterisation of resting cardiac structure and function. Breath-hold steady-state free precession (SSFP) sequences will be used for the quantification of ventricular volumes ventricular function and cardiac mass, whilst non-contrast T1 mapping will be used for myocardial tissue characterisation.

### Central vascular stiffness

Central (aortic) stiffness will be assessed using ECG-gated resting CMR cine-imaging conducted prior to the exCMR. Transverse images of the ascending aorta will be taken just above the sinotubular junction. Cine images will be analysed for changes in 2-dimensional area across the cardiac cycle that can be incorporated with SV (calculated from breath-hold SSFP images) and pulse pressure (obtained from brachial blood pressure measured by an automated cuff) to calculate aortic distensibility and compliance in line with previously validated methods [53].

### Biochemical and blood-based markers

Troponin-I and B-type natriuretic peptide (BNP) will be collected as markers of myocardial injury and myocardial stress respectively. These will be obtained from a non-fasted blood sample taken by a trained phlebotomist 10-min following the exCMR procedure. BNP will be analysed immediately at the Baker Heart and Diabetes Institute using a point of care analyser (Biosite [Alere] Triage MeterPro), whilst an additional sample will be sent immediately to the Alfred Hospital Pathology Laboratory for assessment of troponin-I and haemoglobin. Information related to the use of erythropoiesis stimulating agents or the occurrence of blood transfusion will be obtained from participant medical records. Participants will also have the option of providing ‘opt in’ consent for storing an additional 3.0 mL blood sample for result verification and future analysis of cancer and cardiometabolic-related outcomes.

### Blood pressure

Supine resting systolic- and diastolic BP and resting HR will be assessed from three measurements using an automated machine (OMRON HEM-907, OMRON Corporation, Tokyo, Japan). Measures will be collected after participants have been resting for at least 10-min, with at least 3 min in-between each measurement.

### Total and regional body composition and bone mineral density

#### Dual-energy X-ray absorptiometry

Total and regional fat mass, lean body mass and percentage body fat will be measured from a total body DXA scan (GE Lunar iDXA, GE Healthcare, Little Chalfont, United Kingdom) according to a standardised protocol. Regional composition will be manually assessed using enCore analysis software version 14.10.022 according to standardised procedures. DXA will also be used to quantify areal bone mineral density (g/cm^2^) of the total hip, femoral neck, and lumbar spine (L1-L4 vertebrae).

#### Magnetic resonance imaging

Muscle volume and muscle fat fraction of the quadriceps in the mid-thigh will be assessed by two-point Dixon-based MRI (Siemens Prisma 3 T MRI) conducted immediately prior to the CMR scans. The two-point Dixon method has been validated as an accurate and reproducible (CV = 0.6%) measurement of muscle-fat content [54, 55]. MRI scans of the dominant thigh will be acquired in the supine position, from the superior patella to the greater trochanter. Images will be transferred to a separate workstation for manual off-line analysis of thigh muscle volume and muscle fat fraction (ImageJ2 v1.52d). Muscle volume will be calculated from the summation of disks method by multiplying the sum of the combined regions of interest by the inter-slice distance. Fat fraction will be calculated from the ratio between the fat and combined fat and water signal intensities for the regions of interest.

### Anthropometry

Height and body mass will be used to calculate body mass index and body surface area. Waist circumference will be assessed at the mid-point between the iliac crest and lowest rib according to standard techniques [56].

### Muscle strength

Maximal isometric grip strength will be assessed using a digital grip strength dynamometer (Jamar Plus Digital, Lafayette Instrument Company, IN, USA) following a standardised protocol. Maximal dynamic muscle strength (in kilograms) of the upper body (seated row) and leg muscles (leg press) will be assessed on resistance machines using a 1RM protocol according to current guidelines [57].

### Physical function

Physical function will be assessed using the usual and fast gait speed test, 30-s sit-to-stand test, and timed stair climb. All tests will be performed in triplicate, with the best of the three scores used for analysis.

#### Timed stair climb

The timed stair climb test is a measurement of lower limb muscle power [58]. Participants will be instructed to climb one flight of 12 stairs (17 cm per step) as quickly and safely as possible, using the handrail only if necessary for safety purposes or to regain balance. Stair climb power will be calculated according to the following formula:

$$ \left[\mathrm{weight}\ \left(\mathrm{kg}\right)\mathrm{x}\;9.81\ \mathrm{x}\ \mathrm{step}\ \mathrm{height}\ \left(\mathrm{m}\right)\ \mathrm{x}\ \mathrm{step}\ \mathrm{number}\right]\div \mathrm{time}\ \left(\sec \right) $$

#### Sit to stand test

The 30-s sit to stand test will be used as a measurement of functional lower limb muscle endurance [59]. Participants begin in a seated position (on a chair of standardised height) with their arms folded across their chest. When instructed by the researcher, participants are required to stand fully upright and return to a seated position as many times as they can in 30 s.

#### Gait speed test

The usual- and fast- pace gait speed test is a measure of gait speed and functional mobility [60, 61]. For the usual gait speed test, participants will be required to walk at their usual walking speed between two cones spaced eight metres apart (consisting of a 2 metre acceleration zone, a 4 metre timed zone, and a 2 metre deceleration zone). The time begins when the participant’s front foot enters the timed zone and ends when their front foot enters the deceleration zone. The fast gait speed test is performed in an identical fashion, however in this instance participants are instructed to cover the distance as quickly and safely as possible without running.

### Cognitive function

A series of short verbal and paper-based tests will be used to assess different domains of cognitive function that may be negatively impacted during chemotherapy, including verbal memory, short-term and working memory, and executive function [62].

#### Rey auditory verbal learning test

Changes in verbal memory and learning will be assessed using the Rey Auditory Verbal Learning Test [63]. Outcomes include the total number of words correctly recalled on each attempt, the number correctly recalled after interference, loss after interference (trial 5 minus trial 2) and correct recall after the extended delay period.

#### Digit span test

Short-term and working memory will be assessed using the Digit Span Test [64]. Changes in short-term memory will be assessed using the forwards digit span test and working memory will be assessed using the reverse digit span test. Participants will be scored on the number of sequences recalled correctly for each condition.

#### Trail making test

Changes in executive function will be assessed using the Trail Making Test (Parts A and B) [65].

#### National Adult Reading Test

*The* National Adult Reading Test will be administered to assess verbal, performance and full-scale intelligence quotas as an estimate of premorbid intelligence [66]. Participants are scored based on the number of pronunciation errors. As this test is a measure of premorbid intelligence it will only be administered at baseline.

### Health-related quality of life, fatigue and mood

Health-related quality of life and fatigue will be assessed by the Functional Assessment of Cancer Therapy-Breast (FACT-B) and Functional Assessment of Cancer Therapy-Fatigue (FACT-F) questionnaires respectively, whilst mood will be assessed using the Hospital Anxiety and Depression Scale. All of these questionnaires have been validated for use in cancer patients [67–69].

### Diet, physical activity and sedentary behaviour

#### 24-h food recall

Diet will be assessed by a 24-h food recall completed using the Automated Self-Administered 24-Hour Dietary Assessment Tool (ASA24) [70]. Participants will be prompted to record type and quantity of foods, drinks and supplements consumed over a 24-h period using an Australian-specific food database.

#### Objectively measured physical activity

Habitual physical activity and sedentary behaviour will be objectively assessed over seven consecutive days using hip-mounted ActiGraph GT3X (ActiGraph, Pensacola, FL, USA) and thigh-mounted activPal accelerometers (PAL technologies, Glasgow, Scotland) [71].

#### Self-reported physical activity

Self-reported weekly physical activity over the preceding month will be assessed using the CHAMPS questionnaire [72]. For the baseline assessment participants will be asked to recall their typical physical activity prior to diagnosis.

### Health and treatment-related information

A general lifestyle questionnaire will be used to collect information relating to participant age, health status and medical history, cardiovascular medications and cardiovascular risk profile. Clinical variables related to cancer diagnosis and treatment histopathology, previous and current therapy, chemotherapy regime and treatment response will be obtained from participant medical records. The average relative dose intensity of the originally planned chemotherapy regimen that is received will be calculated according to standard formulae [73], and will be used as a measure of treatment completion. The presence and concentration of tumour infiltrating lymphocytes, in addition to the Miller-Payne grading for patients receiving neoadjuvant chemotherapy will be used to explore the potential for exercise to modulate the tumour response to neoadjuvant therapy.

### ET adherence and attendance

Attendance and adherence to the prescribed number of exercise sessions, and dose within each exercise session (both supervised and unsupervised) throughout the 12-month intervention will be assessed using the Physitrack online patient software which will be logged by participants during each session. Attendance will be calculated from the number of sessions completed versus number prescribed per week. Adherence to the aerobic training will be calculated from the prescribed vs completed duration and intensity of aerobic exercise, whilst adherence to the resistance training will be assessed from the prescribed vs completed repetitions and weight. For the supervised sessions during Phase 1 and 2, trainers will record reasons for modification (increase or decrease) to the prescribed dose of aerobic and/or resistance exercise during each session.

### Adverse events

The occurrence of any adverse events (AEs) will be collected at each testing visit via face to face interview. Participants in the exercise training group will also be asked about the occurrence of an AE at each training session. An event will be considered an AE where there is any possibility that the event related to a study procedure or the exercise intervention. An AE will be classified as a serious AE if it results in death, is immediately life threatening, requires inpatient hospitalisation, requires prolongation of existing hospitalisation, or results in persistent or significant disability/incapacity.

### Data management

Participants will have their personal information de-identified using a code available only to members of the research team. Electronically-stored data will be double entered in a de-identified format onto a secure online data management system (REDCap, Vanderbilt University, Nashville, USA). Frozen blood samples will be kept indefinitely in a re-identifiable format in a − 80 °C freezer. There will not be a formal data monitoring committee for this study, however the study team will meet monthly to review study progress and data will be checked at regular intervals during the study.

### Sample size calculation and statistical analysis

Our sample size of 100 subjects will address Aims 1 and 2 with sufficient compensation for expected attrition of 10% based on our previous pilot work in which two women withdrew (moved interstate and severe treatment related illness) [32]. Primary and secondary analyses will be analysed on an intention-to-treat basis in line with the CONSORT guidelines. Participants who discontinue the intervention will still be asked to attend follow-up evaluations and their results will be included within the intention-to-treat analysis. The significance level for statistical analysis will be set at 5%.

The sample size estimations for Aim 1, that significantly fewer patients undergoing exercise therapy will be functionally disabled at 12 months, are based on our pilot study in which 29% of the total cohort met this criteria at treatment completion and in which there was a 7-fold greater proportion of functional disability in the usual care arm (Usual care: 50% vs Exercise Training: 7%) [32]. In the current proposal, we have used a conservative 24% incidence of functional disability (cf. 29% in the pilot) and three-fold difference between groups (cf. 7-fold in the pilot) [32]. To detect a 36% vs 12% difference in the prevalence of functional disability, 45 women in each group is required (β = 20%, α = 5%). Generalised linear mixed models (GLMMs) with participants as the random effect, time as a repeated measures, and group and group-by-time interactions as the fixed effects, will be used to evaluate the differential effects of the intervention on the incidence of functional disability and additional secondary outcomes. All data will be analysed unadjusted, and adjusted for models including potentially important covariates found to be significantly different between groups at baseline that could explain residual outcome variance. No imputation will be performed for subjects who have missing data due to dropping out of the study. Pre-planned per-protocol analysis including only subjects attending > 66% of the planned exercise sessions will explore the influence of exercise adherence on the primary and secondary outcomes.

The second primary aim of the study is to compare the predictive ability of standard-of-care measures (LVEF) with peak Qc measured by ExCMR in identifying women who will meet criteria for functional disability at 12 months. A multivariate regression will be used with five variables entered (age, LVEF, GLS, study group and peak Qc). The analysis will be stratified for group allocation (usual care vs exercise training) to account for the potential influence of the exercise intervention on the incidence of functional disability at 12-months. The sample size estimated for such an analysis can be calculated as 90 women (50 + 8 x no. of variables) using the method suggested by Green [74].

## Discussion

Given the majority of early-stage BCa patients will be cured, there is a growing focus on minimising the negative effects of cancer treatment on multidimensional health outcomes and quality of life [75]. This is particularly true for AC, which results in excellent cancer-related outcomes, but can cause cardiovascular injury resulting in cardiotoxicity [15] and functional disability [31, 32]. Two major issues that impact on the ability of care providers to minimise these effects are the limited ability to reliably capture patients at risk of subsequent cardiac dysfunction, and a limited evidence on effective and pragmatic preventative therapies [14, 15]. Current cardiac surveillance and risk stratification approaches [14, 15] focus on assessing cardiac function at rest - a condition of low haemodynamic and metabolic stress - which provides little information about cardiac reserve and has weak relationship with other important prognostic markers such as VO_2_peak and functional capacity [31, 32]. This study will provide an important comparison between the current standard of care, and a novel exercise-based assessment of cardiac function which may be more sensitive to cardiovascular injury and functional decline.

This study aims to assess whether exercise training can be offered to at risk women as a means of primary prevention against declines in cardiac function and functional capacity, thereby improving quality of life for longer. Current guidelines for the management of anthracycline-induced cardiotoxicity focus on pharmacological intervention only at a point when patients develop an asymptomatic reduction in LVEF or symptomatic heart failure [14, 15]. However, by this point it is likely that a reasonable degree of cardiac injury has already occurred. Additionally, these medications are likely to have minimal impact on peripheral factors such as skeletal muscle that are also likely to contribute to functional disability. Current guidelines have limited- or generic recommendations to be physically active as part of general healthy lifestyle advice with little evidence base to support these recommendations [14, 15]. Therefore, there is a need for well-designed trials that specifically investigate the role of structured ET as a primary prevention strategy to inform specific exercise- and cardio-oncology guidelines for patients exposed to AC. A handful of randomised trials have assessed the effect of exercise training on cardiac function in small populations of BCa patients receiving AC [40, 43], however they have relatively modest sample sizes, have primarily assessed cardiac function at rest and looked at short-term (12–16 weeks) effects of exercise training on cardiac function and/ exercise/functional capacity. This will be the first study to assess the effect of long-term (12 month) structured exercise training on cardiac function and the clinical endpoint of functional disability in a large population of BCa patients receiving AC. Importantly, this study will be able to quantify the effect of exercise training on cardiac reserve, which is likely to be more sensitive to the beneficial effects of exercise training, whilst also better explaining changes in exercise capacity than resting measures. Importantly, this is one of the few trials to look at the effect of long-term exercise training on fitness and cardiac function. Whilst exercise trials conducted among BCa patients undergoing chemotherapy have shown beneficial effects on preserving VO_2_peak, [40–42] long-term health benefits are more likely if the response can be sustained through the entire treatment trajectory. Given BCa patients are likely to receive a number of additional treatments over the months following AC, [9] this trial will provide important information about whether the benefits of exercise training can persist in the face of the multiple hits imposed by contemporary BCa treatment regimens.

Ultimately, it is hoped that findings from this study will inform clinicians of the relative utility of exercise-based assessment of cardiac reserve for predicting patients at increased risk of cardiovascular and functional decline, whilst also providing evidence for a potentially efficacious preventative therapy in the form of exercise (which is currently recommended as an adjunct therapy, but rarely incorporated into patient care).

### Trial status

At the time of submission this trial is currently recruiting participants.

## Supplementary information

**Additional file 1.**

## Data Availability

The datasets used and/or analysed during the current study are available from the corresponding author on reasonable request.

## Abbreviations

%HRR: Percentage of heart rate reserve
1RM: One repetition maximum
2D: 2-dimensional
3D: 3-dimensional
AC: Anthracycline chemotherapy
AE: Adverse event
BCa: Breast cancer
BNP: B-type natriuretic peptide
BP: Blood pressure
CMR: Cardiac magnetic resonance imaging
CPET: Cardiopulmonary exercise test
DXA: Dual-energy x-ray absorptiometry
ExCMR: Exercise cardiac magnetic resonance imaging
ET: Exercise training
FACT-B: Functional Assessment of Cancer Therapy-Breast
FACT-F: Functional Assessment of Cancer Therapy-Fatigue
GLS: Global longitudinal strain
HF: Heart failure
HR: Heart rate
HR_peak_: Heart rate peak
HER2: Human epidermal growth factor receptor 2
LV: Left ventricular
LVEF: Left-ventricular ejection fraction
MRI: Magnetic resonance imaging
PRT: Progressive resistance training
Qc: Cardiac output
RPE: Rating of perceived exertion
RV: Right ventricular
SSFP: Steady-state free precession
SV: Stroke volume
UC: Usual care
VE: Minute ventilation
VCO_2_: Volume of carbon dioxide production
VE/VCO_2_ slope: Minute ventilation to carbon dioxide production slope
VO_2_peak: Volume of peak oxygen consumption
VT: Ventilatory threshold
