# Supplementary material for: Exercise as a diagnostic and therapeutic tool for preventing cardiovascular morbidity in breast cancer patients– the BReast cancer EXercise InTervention (BREXIT) trial protocol
Source: BMC Cancer. 2020 Jul 14;20:655. doi: 10.1186/s12885-020-07123-6 (PMC7362469; doi:10.1186/s12885-020-07123-6)
Supplement: Supplementary file 1 — Additional file 1. [file 12885_2020_7123_MOESM1_ESM.docx]

**Additional file 1.** Summary of trial registration data

| **Data category** | **Information** |
| --- | --- |
| Primary registry and trial identifying number | Australian and New Zealand Clinical Trials Registry (ACTRN12617001408370) |
| Date of registration in primary registry | 05 October, 2017 |
| Secondary identifying numbers | Alfred Health (Project No: 305/17) |
| Source(s) of monetary or material support | World Cancer Research Fund, Australian National Heart Foundation Future Leader Fellowship, Baker Heart and Diabetes Institute, Deakin University |
| Primary sponsor | World Cancer Research Fund |
| Secondary sponsor(s) | YMCA Victoria; Fitness First; Goodlife Health Clubs |
| Contact for public queries | Mr Stephen Foulkes [steve.foulkes@baker.edu.au] |
| Contact for scientific queries | A/Prof Andre La Gerche [andre.lagerche@baker.edu.au] |
| Public title | Exercise for diagnosis and treatment of heart dysfunction among breast cancer patients |
| Scientific title | Exercise as a diagnostic and therapeutic tool for the prevention of cardiovascular morbidity in breast cancer survivors – a randomised trial |
| Countries of recruitment | Australia |
| Health condition(s) or problem(s) studied | Breast cancer survivors scheduled to undergo anthracycline-based chemotherapy |
| Intervention(s) | Intervention: Multi-modal, supervised exercise training |
|  | Control: Usual care |
| Key inclusion and exclusion criteria | Ages eligible for study: 40-75 years; Sexes eligible for study: Female; Accepts healthy volunteers: No |
|  | Inclusion criteria: Women aged 40-75 years who have been diagnosed with breast cancer and are scheduled to undergo anthracycline-based chemotherapy will be eligible to participate in the study. All eligible participants must also obtain medical approval from their physician before participating. |
|  | Participants will be excluded if they (1) have known structural heart disease (such as symptomatic ischemic heart disease, significant valvular disease or inherited cardiomyopathies), (2) have contraindications to the CMR procedure (pacemakers, implanted metallic foreign body or device), (3) have the presence of any serious contraindication or uncontrolled medical condition that would limit participation in the exercise program, (4) an inability to complete questionnaires in english language or (5) significant cognitive impairment. |
| Study type | Interventional |
|  | Allocation: randomised; Intervention model; parallel assignment; Masking: Open (masking not used) |
|  | Primary purpose: Treatment |
|  | Phase IV |
| Date of first enrolment | November, 2017 |
| Target sample size | 100 |
| Recruitment status | Recruiting |
| Primary outcome(s) | 1. Incidence of functional disability (VO_2_peak ≤18.0mL/kg/min) at 12 months 2. The predictive ability of impaired cardiac reserve (defined as <2-fold increase in cardiac output from rest to high intensity exercise) versus left-ventricular ejection fraction measured at the completion of anthracycline chemotherapy to predict the subsequent development of functional disability (defined as VO2 peak ≤18.0 mL/kg/min) measured at 12 months |
| Key secondary outcomes | Cardiopulmonary fitness; Cardiac reserve and resting cardiac structure and function; Cardiac biomarkers; Vascular function; Body composition; Bone mineral density; Muscle composition; Muscle strength; Functional capacity; Biochemistry; Blood pressure; Cognitive function; Health-related quality of life, fatigue, peripheral neuropathy symptoms, and mood; Habitual physical activity |
